# Factors influencing women’s sex work in a Lebanese sample: results of a case-control study

**DOI:** 10.1186/s12905-020-01062-x

**Published:** 2020-09-05

**Authors:** Maria Slim, Chadia Haddad, Elsa Sfeir, Clara Rahme, Souheil Hallit, Sahar Obeid

**Affiliations:** 1 Faculty of Philosophy and Human Sciences, Lebanese University, Fanar, Lebanon; 2 Research and Psychology Departments, Psychiatric Hospital of the Cross, P.O. Box 60096, Jal Eddib, Lebanon; 3 Faculty of Medicine and Medical Sciences, Holy Spirit University of Kaslik (USEK), Jounieh, Lebanon; 4 Department of Pediatrics, Notre-Dame des Secours University Hospital, Byblos, Lebanon; 5 Faculty of Science, Lebanese University, Fanar, Lebanon; 6 INSPECT-LB: Institut National de Sante Publique, Epidemiologie Clinique et Toxicologie- Liban, Beirut, Lebanon; 7 Faculty of Arts and Sciences, Holy Spirit University of Kaslik (USEK), Jounieh, Lebanon

**Keywords:** Sex workers, Sex work, Child abuse, Partner abuse, Alcohol drinking, Anxiety

## Abstract

**Background:**

Many constituents contribute to the rise of sex work in Lebanon such as the socio-economic situation in the country (poverty, increased unemployment rates, and religious divisions), as well as the political and social instability. Several emotional and psychological factors such as depression, stress, anxiety, low self-esteem, emotional abuse, may force some people to rely on trading sex as a coping strategy for persevering. Therefore, it was deemed interesting to explore and understand factors that are correlated with sex work in Lebanon where no study, to our knowledge, has been written on this critical point. The objective of the study was to assess factors (such as trauma, child abuse, partner abuse, depression, anxiety, and stress) associated with women joining sex work among a sample of the Lebanese population.

**Methods:**

A case-control study was conducted on a group of women (60 sex workers recruited from a prison for women) involved in sex work matched for age and sex with a control group (60 non-sex workers). Controls were chosen from the same prison population as the sex workers.

**Results:**

A logistic regression was conducted, taking being a sex worker vs not as the dependent variable; independent factors were sociodemographic characteristics, child (psychological, neglect, physical and verbal) and inter partner violence (physical and non-physical), depression, anxiety and stress. Higher anxiety (aOR = 1.08) and higher inter partner physical violence (aOR = 1.02) were altogether related with higher chances of being a sex worker.

**Conclusion:**

This study proposes an association between child abuse, inter partner violence, alcohol consumption, anxiety, and sex work. Future research may also need to contemplate other factors not examined here, including parental substance use, personality traits, and many others.

## Background

Sex work is one of the ancient occupations in human history [1]. It comprises a wide range of activities that cater to humans’ natural urges for sex and the desire for money [2]. Sex job is the act of sexual activity, in exchange for money [3]. Further, Kesler defined sex work as serving men’s sexual needs and meeting women’s economic needs [4]. It has been observed that a majority of sex workers are female, and they can have clients of any gender or sexual orientation [5]. A client who buys sex expects the sex worker to provide them with sexual enjoyment, often without caring in the least about the sex worker’s own satisfaction. Different types of sexual services exist around the world but they can be broadly classified into two groups: direct and indirect sex work [6]. Direct sex work refers to indoor work (at a client’s residence or hotel room) or outdoor services such as street and escort sex work [6]. It involves the exchange of sex in lieu of payment, during which genital contact is normal. Indirect sex work (in which genital touch is less frequent) applies to activities such as lap dancing, stripping, and electronic sex activities (online or over the phone). For this sort of sex work a fee for the service is always charged [6].

Stigma is described as “a social trait or mark which distinguishes individuals from others on the basis of judgments provided in society” [7]. This has been found to have a detrimental effect on the formation of self-conception and personality, culminating in degrees of social isolation varying from trouble engaging in regular social activities owing to insecurity or shame to complete ostracism or rejection by others [8]. Sex workers are frequently faced with unacceptable, inappropriate or completely threatening situation due to stigmatizing perspectives negatively influencing their job environment, interactions, and health [9]. They experience several forms of stigma such as disrespect, devaluation, and even violence [9], and it is especially valid for women sex workers, who are often exposed to negative attitudes (harassment, humiliation, and so on.) [10, 11] from the larger community and degrading treatment in public spaces [12]. Moreover, several states criminalize the provision of sexual facilities on the basis that it interferes with the normative framework of a society [13, 14].

A wide range of factors may lead a woman to become a sex worker. Family factors can play a key role given the lack of family support, effective support, family security, as well as separation of parents, violence (sexual and physical abuse), breakdown or absence of close parental supervision and living in a corruptive family environment encourage sex work [15]. Furthermore, Childhood abuse may trigger structural changes in the brain, ultimately impacting social health and relationship development throughout adulthood, contributing to increased susceptibility to violence [16]. Also, intimate partner violence (physical, emotional, verbal or sexual) could encourage sex work. The consequence for such abuse for the victim may vary, often including physical disabilities, depression, low self-esteem, fear and chronic health problems. Some of the sex workers had been victims to sexual, physical and verbal abuse when they were young, thus, their conception about sex had been derailed due to their childhood experiences [17]. The levels of child abuse amongst sex workers vary from 46 to 75% [18–21]. A research performed on a sample of 854 female prostitutes (working on and off the roads) from nine various countries showed that, on average, 63% of the sampled women have experienced sexually abused as a child, with an average of four offenders, while 59% reported being physically abused during childhood by a parent [22]. According to another study conducted on 325 sex workers and drug users, 44.9, 50.5 and 61.8% were victims of physical, sexual and emotional violence during adolescence, respectively [23]. Neglect was also a prevalent characteristic of these women’s home lives, with 58.5 and 45.2% emotional and physical neglect recorded respectively [23].

In addition, the presence of a range of emotional and psychological causes, such as depression, tension, anxiety, low self-esteem, mental violence, will build a negative spiral for many individuals where they find themselves relying on trading sex as a coping strategy for surviving [24]. Sex workers frequently suffer high levels of violence and negative societal views, which make them vulnerable to psychosocial challenges. The latter might predispose them to lose their identity and have low self-esteem and self-worth [25]. These situations make them socially isolated and vulnerable to specific psychiatric illnesses such as anxiety, mood and post-traumatic stress disorders [19, 26]. The prevalence of depression in sex workers vary from 4.2 to 84% [26–28]. In a study done among 692 sex workers, participants had reported that 48.8% of them had been given a diagnosis of mental issue. Depression (35.1%) and anxiety (19.9%) were the most common diagnoses [29]. Furthermore, drug addiction has a direct relationship with sex work as demonstrated in several studies [30–33]. Women having a life full of stress and problems might seek drugs to tolerate life’s pressure and might resort to sex work when they are not able to meet the cost of their addiction [34]. On the other hand, many sex workers can shift to substance use as a coping mechanism for the challenging lifestyles associated with sex work. In addition, drug addiction by sex workers might be due to the coercion of substance use by someone else. Some sex workers are forced to take drugs with either the sex club owner or the client who shares his/her drugs with the worker in order to further enjoy the night [35].

Lastly, the low socioeconomic status is considered an essential factor that leads to women being involved in sex work [36]. Loans, unemployment, poor education, health issues, household troubles, crime, absence of social assistance and other detrimental events in life encourage a woman to continue sex work [37]. Moreover, women also engage in sex work to earn money to survive especially when they cannot seem to find other jobs and opportunities of employment due to the lack of qualifications and adequately paid jobs [6].

Although human rights for sex workers are rarely considered, equality and protection from violence remain the key rights of every female sex worker [38]. Sex work exists in every society; it has been legitimized in some of them [39], but impermissible and unregulated in others. The aspect of sex work in societies varies; in some societies, people view sex work as an expression of sexual liberation, whereas in others, it is considered a violent act against women and a public health issue [39]. In the two contrasting theoretical views, the neo-abolitionist group opposes all aspects of illicit and unintended prostitution as acts of abuse against women, and asserts that prostitution is never legal. The other party, including several sex positivists, thinks a woman has the freedom to pursue prostitution and other forms of sex work as a means of jobs or as an occupation [40].

The majority of the Middle East countries prohibit prostitution but do not have specific prostitution laws [41]. Two Middle Eastern countries do not have an outright prohibition against prostitution: Israel and Lebanon [41]. Lebanon, however, has specifically legalized prostitution for women, but not men [41]. In Lebanon, sex work is legal and regulated, however, no official licenses are given to the places where such services are practiced [42]. Super night clubs in Lebanon have a semi-official license, with sex work being illegally done in the streets, bars, hotels, and brothels [42]. Further, Lebanon is an input and output country for sex-trafficked women and children and a gateway route for Eastern European women and children who are exposed to sex-trafficking in other middle east nations. Females from Eastern Europe and North Africa join Lebanon to operate in the adult entertainment sector via the performer visa scheme in Lebanon, which retains a significant commercial sex industry and allows for sex trade [43]. Many factors contribute to the rise of sex work in Lebanon such as the socio-economic situation in the country (poverty, increased unemployment rates, and religious divisions), as well as the political and social instability [44]. Furthermore, the Syrian crisis resulted in a flow of refugees, which has aggravated the problems faced by the Lebanese population, particularly the poor people [44], and their xenophobic attitudes towards immigrants [45]. In this context, Most women and girls from Syria were hired on false job promises and forced to submit to prostitution where they encountered mental, physical and sexual mistreatment and forced abortions [43]. In addition, violence is another core problem of the Lebanese patriarchal society where women are victims of rape and forced sexual relations. These reasons had forced many women to involve in sex work whether they were Lebanese, Syrian or from other nationalities [44]. Therefore, it was deemed interesting to explore and understand the factors associated with sex work in Lebanon where no study, to our knowledge, has been written on this critical point. The research aimed to evaluate factors (such as child abuse, inter partner abuse, depression, anxiety and stress) associated with women’s sex work among a Lebanese sample.

## Methods

### Reaserch design

A case-control study was carried out on a group of females (60 sex workers recruited from prison) involved in sex work matched for age and sex with a control group (60 non-sex workers). Interviews were done upon arrival (within 2–3 days) of the sex workers and the controls to prison, anonymously. Permission to approach each participant was obtained from the person in charge of the prison. Afterward, participants were asked to enroll in the study, with no financial rewards offered to them for participation. Psychological help was available in prison but did not influence the interview process. Information about such activity was retrieved from their file in prison and as per the local police/prison guards; sex workers started their activity within a month or less before starting the data collection. Controls were chosen from the same prison and they self-reported not being sex workers; this information was verified using the psychological report included in the file of each prisoner. The control group will allow us to establish the correlation of factors with the engagement of women in sex work. The research surveyed the first qualifying person who agreed to engage in the research. Those applicants above the age of 18 have the right to be involved. Individuals who refused to take part in the study have been excluded. One qualified employee, a counselor, was in charge of data gathering, via a face-to-face informal consultation with each participant. That person had been independent of the report.

### Questionnaire

The survey used during the interview was in Lebanon’s mother language, Arabic. First part evaluated the participants’ sociodemographic features (age, gender, educational level, marital status, employment status, monthly income (divided into four categories: no salary, low salary < USD 1000; moderate salary of USD 1000–2000; and high salary > USD 2000). Variables of alcohol and substance use within the last month were dichotomized (yes/no). The other sections contained the different measures used in this analysis that assessed child or partner abuse at any time during their lifetime, as well as depression, anxiety and stress symptoms within the last month.

### Child abuse self report scale (CASRS)

The CASRS measures on a Likert basis (0–3 point each) four types of child abuse, psychological (14 items), neglect (11 items), physical (8 items), and sexual (5 items) [46]. The higher the scores, the higher the child abuse. In this research, Cronbach’s alpha for the four types of child abuse were 0.935 for psychological abuse, 0.905 for neglect abuse, 0.944 for physical abuse, and 0.925 for sexual abuse.

### Composite abuse scale

This scale measures physical and non-physical abuse in a relationship between partners respectively. This 15-item scale [47], scored on a Likert scale of seven-point ranging from 0 (not in the past 12 months) to 5 (daily/almost daily). The higher the ranking, the greater the violence / abuse between spouses. The Cronbach’s alpha values for both physical and nonphysical subscales were 0.991.

### Hamilton depression rating scale (HDRS)

The validated Arabic version of the HDRS was used in this study [48]. The first 17 items measure the severity of depressive symptoms. Higher scores indicate more severe depression. The Cronbach’s alpha of the HDRS was 0.940 in this study.

### Hamilton anxiety scale (HAM-A)

The HAM-A [49], validated in Lebanon [50], comprises 14 items, rated from 0 (no symptoms) to 4 (very severe symptoms) on a Likert scale of five points. High ratings are indicative of greater anxiety. In this study, the Cronbach’s alpha of the HAM-A was 0.955.

### The perceived stress scale (PSS)

The ten questions in this scale ask about the last month’s feelings and thoughts, with the answers measured on a Likert scale of five points: 0 (never) up to 4 (very often). Higher scores represent the greater tension perceived. In this study, the Cronbach’s alpha was 0.810 for this scale.

### Statistical analysis

Version 23 of the SPSS program was used for the data analysis. For reliability analysis of each scale / subscale, Cronbach’s alpha values have been registered. The Student t-test was used to compare continuous variables between the two groups. The exact measures of the chi-square and the Fisher were used to evaluate categorical variables. All variables displaying a *p* < 0.1 in the bivariate study were regarded as significant variables to be included in the final model to exclude any confounding factors [51]. Multiple models of forward logistic regression were used to evaluate the mediating effect of alcohol between all variables (stress, anxiety score, depression, child and inter partner violence/abuse) and sex work. Socio-demographic features were entered as independent variables in the first model, while in the second, child and partner abuse variables were added. Moreover, stress, depression, and anxiety were added in the third model while alcohol use in the fourth model, was introduced as an independent variable. Significance was set to *p* < 0.05.

## Results

### Sociodemographic and other characteristics of the sample population

Details regarding sociodemographic and other characteristics of the participants are shown in Table 1. The mean age of the participants was 30.72 ± 9.21 years. A significantly higher proportion of non-sex workers had a university degree (73.3% vs. 28.3%) compared to sex workers. Also, a significantly higher proportion of sex workers was illegal substance users (36.7% vs. 11.7%) and alcohol users (53.3% vs. 26.7%) compared to non-sex workers.

**Table 1:** Sociodemographic and other characteristics of the sample

	Non sex worker ***N*** = 60 (50%)		Sex worker ***N*** = 60 (50%)		***P***-value
	Frequency	%	Frequency	%	
**Education level**					
Illiterate	3	5.0%	13	21.7%	**< 0.001**
Primary	2	3.3%	13	21.7%	
Complementary	6	10.0%	7	11.7%	
Secondary	5	8.3%	10	16.7%	
University	44	73.3%	17	28.3%	
**Personal monthly income**					
< 1000 $	43	71.7%	51	85.0%	0.094
1000$ - 2000$	15	25.0%	6	10.0%	
> 2000 $	2	3.3%	3	5.0%	
**Marital status**					
Single	58	96.7%	57	95.0%	0.843
Married	1	1.7%	2	3.3%	
Widowed	0	0.0%	0	0.0%	
Divorced	1	1.7%	1	1.7%	
**Work status**					
Unemployed	45	75.0%	51	85.0%	0.171
Employed	15	25.0%	9	15.0%	
**Substance use**					
Yes	7	11.7%	22	36.7%	**0.001**
No	53	88.3%	38	63.3%	
**Alcohol use**					
Yes	16	26.7%	32	53.3%	**0.003**
No	44	73.3%	28	46.7%	

### Bivariate analysis

**Fig. 1:**
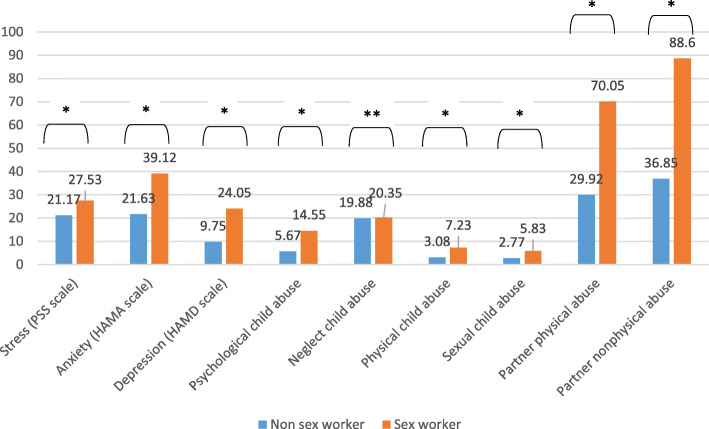
Mean difference of the used scales between non sex-worker and sex worker. *p < 0.05, **p > 0.05

Higher mean stress (PSS scale) (27.53 vs. 21.17), anxiety (HAMA scale) (39.12 vs. 21.63), depression (HAMD scale) (24.05 vs. 9.75), psychological child abuse (14.55 vs. 5.67), physical child abuse (7.23 vs. 3.08), sexual child abuse (5.83 vs. 2.77), partner physical abuse (70.05 vs. 29.92) and partner nonphysical abuse (88.60 vs. 36.85) scores were significantly found in sex workers compared to non-sex workers (Fig. 1).

### Multivariable analysis

A set of logistic regressions was conducted taking being a sex worker vs not as the dependent variable. The results of a first model, taking the sociodemographic characteristics as independent variables, showed that having a university education level (aOR = 0.09) compared to illiteracy was significantly associated with lower odds of being a sex worker (Table 2, Model 1).

**Table 2:** Multivariable analysis: Logistic regression taking non-sex worker^a^/sex worker as the dependent variable

**Model 1: Independent variables: Sociodemographic characteristics**				
**Variable**	***p*-value**	**ORa**	**95% CI**	
**Education level**	**< 0.001**			
Illiterate		1		
Primary	0.683	1.50	0.21–10.52	
Complementary	0.122	0.27	0.05–1.42	
Secondary	0.359	0.46	0.09–2.41	
University	**0.001**	0.09	0.02–0.35	
**Model 2: Independent variables: Sociodemographic characteristics and child and partner abuse.**				
**Variable**	***p*-value**	**ORa**	**95% CI**	
**Education level**	**0.031**			
Illiterate		1		
Primary	0.907	0.88	0.10–7.45	
Complementary	0.266	0.35	0.05–2.25	
Secondary	0.116	0.21	0.03–1.47	
University	**0.008**	0.13	0.03–0.59	
Partner non-physical abuse	**< 0.001**	1.03	1.02–1.05	
**Model 3: Independent variables: Sociodemographic characteristics, alcohol use, child and partner abuse and stress, depression and anxiety.**				
**Variable**	***p*-value**	**ORa**	**95% CI**	
Anxiety (HAMA scale)	**0.002**	1.08	1.03	1.13
Partner physical abuse	**0.004**	1.02	1.01	1.04

Hosmer and Lemeshow test (*p* = 0.745); Nagelkerke *R*^2^ = 0.617

Variables entered in the final model: education level, socioeconomic status, stress, anxiety, depression, psychological child abuse, physical child abuse, sexual child abuse, partner physical abuse, partner nonphysical abuse, alcohol use and substance use

^a^ Reference group; numbers in bold indicate significant *p*-values

The results of a second model, taking the sociodemographic characteristics and child (psychological, neglect, physical and verbal) and physical and non-physical inter partner violence as independent variables, showed that higher inter partner non-physical violence (aOR = 1.03) was significantly associated with higher odds of being a sex worker (Table 2, Model 2).

The results of a third model, taking the sociodemographic characteristics, child (psychological, neglect, physical and verbal) and inter partner (physical and non-physical) violence/abuse scores, as well as depression, anxiety, and stress as independent variables, showed that higher anxiety (aOR = 1.08) and higher inter partner physical abuse (aOR = 1.02) were significantly associated with higher odds of being a sex worker (Table 2, Model 3).

In a fourth model, when adding the variable alcohol use to the third model to test its mediating effect on all other variables, the results remained the same.

We then analyzed the interaction between alcohol and all other variables (stress, anxiety score, depression, psychological child abuse, sexual child abuse, physical child abuse, partner physical and non-physical abuse). The multivariable analysis results did not differ from the ones obtained in the third model.

It is noteworthy that the other variables (monthly income, education level, and substance use) were eliminated by SPSS from the final model; thus, the final results were considered to be adjusted to those variables.

## Discussion

Determination to satisfy everyday needs is one of the major factors described as a predisposing factor for sex work [52]. However, it is not the only reason females choose this type of job. This study revealed that higher rates of anxiety and partner physical abuse were associated with sex work, but are independent of alcohol use.

### Anxiety

As far as we know, there is no study conducted in Lebanon assessing the prevalence of mental health problems and sex work. Our study is the first to reveal that anxiety was associated with higher odds of pursuing this type of job in agreement with literature. Indeed, anxiety is one of the most common mental health problems found in female sex workers [29, 53, 54]. Even though these female workers have increased mental health problems, their prevalence is variable in international literature. In fact, 19.9% of sex workers in Canada reported having anxiety [17]. Simons et al. discovered, in a study conducted on 193 female sex workers in Zurich, that anxiety’s effect on these women was 34% [55, 56]. Similarly, a study conducted in Iran showed that 39% of sex workers suffered from anxiety, while these rates in Austria were 17% and reached 31% in Israel [56]. Some female sex workers mentioned that they experienced neglect in their childhood and that carelessness influenced their personality development [57]. They developed inclined personalities with a desire to protest against any demand that makes them adhere to rules. All those circumstances placed pre-sex women workers in a state of continuous anxiety. In fact, sex work is, according to these women, a method used to escape their long-lasting anxiety [54, 58].

### Physical abuse

Female sex workers are prone to sexual abuse, either from their clients or partners [59]. A study conducted in China showed that 58% of female sex workers experience violence from their stable partners [60]. Furthermore, our study found that Lebanese female sex workers have higher rates of partner physical and sexual abuse, in comparison to non-sex-worker females. Another study showed 36.9% of female sex workers suffer from partner physical abuse and are associated with higher rates of mental health problems [59]. Higher rates of sex work were found in African female sex workers facing intimate partner violence [61, 62]. In fact, a study conducted in India on female sex workers showed that many young married girls are beaten and abused. This sexual and physical exploitation could be a motive for being a sex worker [61, 62]. The emotional stress and the resulting mental health illnesses faced by women can consequently influence their choice of becoming a sex worker. For example, many female sex workers that face sexual or physical molestation will escape their homes to avoid and stop the assaults and will practice sex work to regain control over their sexuality [58].

### Alcohol use

Alcohol use was associated with higher odds of being a sex worker among Lebanese women in the bivariate, but not the multivariable, analysis and did not mediate the association between all other variables and sex work. This might be due to the small sample enrolled in this study and to the fact that alcohol use was assessed using a single question (not through a validated scale) with a self-reported answer. Many studies have shown that early exposure to alcohol can be a predisposing factor to becoming a sex worker [56, 63]. In addition, many studies relate the higher rates of female workers’ alcohol addiction to the fact that it is a coping mechanism used to deal with economic problems and stressful work [64]. Furthermore, substance abuse, including alcohol, helps female sex workers that we are morally opposed to entering sex work violate their beliefs to support their habits [65].

Our study showed that sex workers were mostly unemployed and had a low socioeconomic status. Similarly, previous findings conducted on female sex workers showed the same descriptive characteristics [23]. Many interviewed female sex workers declare that sex work generates income. From an economic perspective, sex work can help them get items they were not able to afford before joining this work field [23, 25, 26]. Besides, most sex workers in our sample were single; in fact, sex work was considered an escape from a toxic relationship [28].

## Clinical implications

Our study adds useful information about sex work in Lebanon and shows similar results to international findings. In addition, our findings highlight many preventable factors that can lead women to sex work. Moreover, more interventional and preventive measures should be taken in order to address sex workers’ needs. Focusing on treating physically and sexually-assaulted women, as well as protecting adolescent girls at risk, can be a helpful method to decrease the chances of these women joining this work field. Also, implementing strategies to preserve women’s dignity and help them find legal jobs are other ways to reduce sex work. On the other hand, psychological and psychiatric support can help decrease alcohol use/abuse. It is important to say that decreasing sex work among women does not only have social and psychological benefits but can also be a lifesaving method since studies have shown high burdens of sexually transmitted diseases in female sex workers [62].

## Limitations and strengths

Our study has many limitations. First of all, the sample size is small and our results cannot be extrapolated to the general population; however, we believe that the results are reliable and have enough statistical power. Also, being a case-control study, a causal relationship between the factors cited and sex work cannot be determined. Furthermore, social desirability biases are also possible, with the temptation of female sex workers to make a subjective link with the mentioned factors to explain their condition. The fact that sex workers and controls were recruited from prison might cause a selection bias and information bias since imprisoned people may be more prone to mental health problems (depression, anxiety, stress, etc.). Moreover, other variables could have altered the outcome (coercion, underage entry, sex work in the family, etc.), which were not taken into consideration in this study. Therefore, a longitudinal study is needed to assess risk factors associated with becoming a sex worker in Lebanon, while considering the results of this study. One major strength of this study is the case-control design, which is used to study the rare outcomes. This design is usually used to create suggestions for further prospective studies.

## Conclusion

This study suggests an association between partner abuse, anxiety, and sex work. Future research may also need to consider other factors that were not examined here, including parental substance use, personality traits, and many others. In addition, sex work can predispose women to mental and health problems that can be life-threatening and have lifelong morbidity. Hence, further studies are needed to assess mental health problems in Lebanese sex workers after facing the job’s stress.

## Data Availability

All data generated or analyzed during this study are not publicly available to maintain the privacy of the individuals’ identities. The dataset supporting the conclusions is available upon request to the corresponding author.

## Abbreviations

HDRS: Hamilton depression rating scale
CASRS: Child abuse self report scale
HAM-A: Hamilton anxiety scale
PSS: Perceived stress scale
